# Mapping of the Sex Determining Region on Linkage Group 12 of Guppy (*Poecilia reticulata*)

**DOI:** 10.1534/g3.119.400656

**Published:** 2019-09-24

**Authors:** Lior Dor, Andrey Shirak, Yair Yaacov Kohn, Tal Gur, Joel Ira Weller, Dina Zilberg, Eyal Seroussi, Micha Ron

**Affiliations:** * Robert H. Smith Faculty of Agriculture, Food and Environment, Hebrew University of Jerusalem, Rehovot 76100, Israel,; † Agricultural Research Organization (ARO), Volcani Center, Institute of Animal Science, HaMaccabim Road, P.O.B 15159, 7528809, Rishon LeTsiyon, Israel,; ‡ Central and Northern Arava Research and Development D.N. Arava Sapir, 86825 Israel, and; § The French Associates Institute for Agriculture and Biotechnology of Drylands, Ben-Gurion University of the Negev, Midreshet Ben-Gurion, Israel

**Keywords:** *Poecilia reticulata*, sex determination, sex specific marker, mapping, Genetics of Sex

## Abstract

*Poecilia reticulata* is one of the most popular ornamental fish species with a higher demand for males due to their large colorful fins. The objectives of this study were mapping of the sex-determining (SD) region on linkage group 12 of guppy, and identification of a sex specific marker. We generated eight polymorphic microsatellite markers distributed along the distal 5.4 Mbp sequence of the previously identified SD region on linkage group (LG) 12. The markers were tested for association with sex in 156 individuals of the Red Blonde and Flame strains, and 126 progeny of four full-sibs Red Blonde families. A male-specific allele was found for microsatellite gu1066 at position of 25.3 Mbp on LG12 for both strains, and gu832 at position 24.4 Mbp for the Flame strain. Thus, a region of 0.9 Mbp between these markers, harboring 27 annotated genes, was selected for analysis. Based on association of copy number variation and sex determination we mapped a duplicated region between LGs 9 and 12, of 1.26 Mbp, containing 59 genes on LG12. The common interval between the segment bounded by gu1066 and gu832, and the duplicated region of 0.43 Mbp on LG12 harbors 11 genes of which 6 have multiple copies (54%). Growth arrest and DNA damage inducible gamma-like (*GADD45G*-like) is a plausible positional and functional candidate gene for its role in male fertility. We characterized the genomic structure of the gene in guppy, and found two isoforms; but no sex-biased differences were evident in genomic sequence and gene expression.

There are over 34,000 fish species according to FishBase (www.fishbase.de), but only a few hundred species have been studied for environmental, epigenetic and genetic effects on SD (Devlin and Nagahama 2002; Mank *et al.* 2006). In many cichlid species of the genus *Apistogramma*, SD is affected by temperature (Ospina-Álvarez and Piferrer 2008). Epigenetic mechanisms have also been suggested to affect the establishment and maintenance of the gonad differentiation pathways of the half-smooth tongue sole (*Cynoglossus semilaevis*) (Chen *et al.* 2014; Martínez *et al.* 2014). Genetic mechanisms affecting SD include XX/XY sex chromosome system, with the male being heterogametic in medaka (*Oryzias latipes*), Nile tilapia (*Oreochromis niloticus*) and rainbow trout (*Oncorhynchus mykiss*) (Matsuda *et al.* 2002; Eshel *et al.* 2014; Yano *et al.* 2012), or ZZ/ZW system with female being heterogametic in Blue tilapia (*Oreochromis aureus*) or in the half-smooth tongue sole (Don and Avtalion 1988; Chen *et al.* 2008).

In some species like the half-smooth tongue sole or in the Bordallo (*Squalius carolitertii*) there are distinct sex chromosomes (Chen *et al.* 2008; Collares-Pereira *et al.* 1998), while in other species there are no observed sex chromosomes in the karyotype. In medaka and in the Patagonian pejerrey (*Odontesthes hatcheri*) the master sex determining (MSD) genes *e.g.*, *DMY* and *AMHY* are male-specific duplications of regular *DMRT1* and *AMH* on different chromosomes, respectively (Matsuda *et al.* 2002; Nanda *et al.* 2002; Hattori *et al.* 2012). However, in chicken and Nile tilapia *DMRT1* and *AMH* duplications are positioned in a tandem manner (Smith *et al.* 2009; Eshel *et al.* 2014; Li *et al.* 2015). The central role of *AMH* duplication in SD was also demonstrated for Lingcod *(Ophiodon elongates*) (Rondeau *et al.* 2016) and Northern pike (*Esox lucius*) (Pan *et al.* 2019). *SRY* is a mammalian MSD gene that may have evolved from *SOX3* (Waters *et al.* 2007). *SOX3* was detected as an MSD gene in medaka-related fish (*Oryzias dancena*) (Takehana *et al.* 2014). Nevertheless, in many taxonomic groups of animals the duplication of one of the six MSD genes *e.g.*, *DMRT1*, *AMH*, *SOX3*, *AMHR2*, *GSDF* and *IRF9* initiates the SD pathway (Matsuda 2018; Li and Gui 2018). Thus, it was hypothesized that the molecular pathway responsible for SD is conserved among animals, and the MSD gene in the critical SD region of guppy could be deduced based on mammalian studies and human disorders (Shirak *et al.* 2006; Warr and Greenfield 2012; Cutting *et al.* 2013). This approach has been successfully used in our previous study of *AMH* duplication detection in the sex determining region of Nile tilapia (Shirak *et al.* 2006; Eshel *et al.* 2010, 2012, 2014).

The guppy (*Poecilia reticulata*) is a small viviparous poeciliid native to small clear streams in northeastern South America and adjacent islands (Endler 1995). Like other live-bearing species, guppy presents a marked sexual dimorphism in which the male body is much more pigmented and with larger fins than females, with almost continuous color pattern polymorphism (Petrescu-Mag and Bourne 2008). This species is one of the most popular ornamental fishes with a great importance in the global tropical fish trade market (Monticini 2010). However, there is a greater demand for males rather than females, due to their colorful pigmentation (Basavaraja *et al.* 2012). Fish are sorted by hand according to sex identification; which is time consuming, expensive and stressful; thus resulting in sickness and mortality. Our long-term objective is to produce genetically all-male populations. Guppies were characterized with an XY sex chromosome system (Winge 1922). Coloring pattern and fin-shape polymorphism are inherited in a sex linked manner (Winge 1923; Kottler and Schartl 2018). Guppy sex chromosomes are morphologically distinct, containing a pseudo autosomal and unique Y non-recombining region. Thus, the Y chromosome is significantly larger than the X chromosome in most populations of Endler’s guppy (*Poecilia winger*) (Nanda *et al.* 2014) with less pronounced difference in Trinidadian guppy (*Poecilia reticulata*) (Morris *et al.* 2018). Although the characterization of XY sex-chromosome system in guppies was determined some 100 years ago (Winge 1922) the MSD gene is still unknown. The SD regulating genomic region has been identified on linkage group (LG) 12 to its distal end between 22 to 25 Mbp (Tripathi *et al.* 2009; Wright *et al.* 2017).

Feeding of pregnant dams by estrogens 5-10 days before parturition resulted in all-female progeny (Kavumpurath and Pandian 1993). In order to distinguish between sex reversal and natural females, a sex specific marker is required. However, no genetic marker specific for the Y-chromosome and common to all guppy strains has been identified (Kottler and Schartl 2018). The objectives of this study are: 1. mapping of the sex determining region on linkage group 12 of Guppy Red Blonde and Flame, and 2. identification of a sex specific marker that will be used to identify sex reversed females as genetically males for mating with normal males in order to derive YY males.

## Material and Methods

### Guppy strains and families

All fish were maintained and handled in the aquaculture department at the Central and Northern Arava Research and Development center (Israel). Four full-sib families of the Red blonde line (RB) (obtained from Ginat ornamental fish farm, Moshav Ein-Yahav, Arava, Israel) were produced as follows: females were detected by sex specific features such as pigmentation, fin to body size ratio, body size and shape, gravid spot/gonopodium and behavior at about 3 months post-spawning. Each female was separated and raised in a separate tank, and later a single male was added to the tank. The fingerlings of every family were raised in separate tanks until reaching maturity, then sex was determined and the fish were fin-clipped for DNA extraction. In addition, five batches of Red Blonde and Flame strains (obtained from Ginat ornamental fish farm and Manor farm, Hatzeva, Israel) from three different ornamental fish farms in Hatzeva Israel were used for association study of microsatellites with sex (Table 1).

**Table 1:** Distribution of genotypes of males and females by gu1066 genotypes in the Red Blonde (RB) and Flame (FL) lines from 3 farms, and in 4 full-sibs families of Red Blond from the Arava research center

Farm*^a^*	Line*^b^*	Batch/ Family*^c^*	Males		Females		Nominal probability*^d^*	Experiment-wise probability*^e^*
			XY	XX	XY	XX		
Ginat	FL		23	0	0	23	5.7E-10	5.1E-09
Ginat	RB	1	23	0	0	23	5.7E-10	5.1E-09
Ginat	RB	2	6	4	0	10	1.5E-02	NS
Manor	RB		8	2	0	10	3.5E-03	3.1E-02
Ran	RB		6	4	0	10	1.5E-02	NS
Arava	RB	A	9	4	0	3	1.8E-01	NS
Arava	RB	B	20	3	0	15	4.5E-06	4.0E-05
Arava	RB	C	16	2	0	21	5.5E-07	4.9E-06
Arava	RB	D	11	2	0	17	3.6E-05	3.2E-04
Total			122	21	0	132		

a Genotypes of gu1066 were designated “XY” and “XX”, where “Y” is male specific allele of 212 bp (206 or 212 bp in Manor), and “X” - all other alleles.

b FL - Flame; RB - Red Blonde.

c Full-sibs families A to D.

d Significance values of Chi-squared test for association between gu1066 segregating microsatellite and sex, based on detection of male-specific allele of gu1066.

e With application of the Bonferroni correction for multiple comparisons, NS - Not significant.

### DNA extraction and amplification

DNA was extracted from fins or whole body samples using the MasterPure DNA Purification Kit (Epicentre Biotechnologies, Madison, WI, USA) following the manufacturer’s recommended protocol. DNA concentration was quantified using a NanoDrop 1000 Spectrophotometer, and DNA samples diluted to 20 ng/µl were distributed into 96-well PCR plates. Microsatellites were amplified by a two-step PCR following Dor *et al.* (2014), except that the annealing temperature was 59° and 61° for the first and second PCR steps, respectively. After detection of an association between marker gu1066 and SD, a specific fluorescently-labeled forward primer was developed for gu1066 and used in one step PCR amplification with annealing temperature of 61°.

### Marker development

A previous study indicated the localization of a sex determining region of *P. reticulata* in the terminal region of LG12 (Tripathi *et al.* 2009). Therefore, the last 5.4 Mbp sequence of female LG12 was downloaded from NCBI (assembly GCF_000633615.1). The sequence was masked for repeats with CENSOR (Kohany *et al.* 2006) and Tilapia Repeat Masker (Shirak *et al.* 2010) and scanned for the following microsatellite repeat motifs: (CA)_9_NNN/(AC)_9_NNN, (AT)_10_NNN/(TA)_10_NNN, (TG)_10_NNN/(GT)_10_NNN. Evenly spread motifs along the sequence were selected as potential markers and primers were designed using Primer3 (Untergasser *et al.* 2012). Marker names were designated “gu” followed by a three or four-digit number, *e.g.*, gu001. The primer sequences are presented in Supplemental Table S1.

### Genotyping of microsatellite markers

The fluorescently labeled PCR products were detected by an ABI3130 Genetic Analyzer and automatically genotyped by GeneMapper software v.4.0 using GeneScan-500 LIZ size standard (Applied Biosystems, Foster City, CA, USA).

### Statistical analysis

Chi squared test was used to test for divergence of sex distribution from equality between SD genotypes. Fisher’s exact test was computed using PROC FREQ in SAS (version 9.2; SAS Institute Inc., Cary, NC) for association study of microsatellites and sex. The nominal probability of significance was adjusted to account for multiple comparisons using the Bonferroni correction by dividing the nominal probability by the number of comparisons (Hochberg 1988).

### Genomic analysis

Using Ensembl with Biomart option (www.ensembl.org) and NCBI, the female guppy genome was searched for all annotated genes in the genomic region on LG12 between markers gu832 and gu1066. Genes without annotation in Guppy were Blastp searched against “Teleostei”. After detection of similarity between a region on LG9 and the SD region on LG12 duplicate genes on both LGs were identified.

Based on medaka as a reference genome, we searched for duplicated genes on LG9 that belong to the group of six MSD genes or their interacting genes. Boundaries of the region were marked if no additional duplicate genes were identified in the marginal 1 Mbp sequences. The gene list was plotted using MapChart software (Voorrips 2002).

In view of the partial *GADD45G*-like annotation for *P. reticulata*, the *P. formosa* sequence of scaffold NW_006800603.1 (from 35,000 to 41,000 bp) was downloaded and masked using GIRI (Kohany *et al.* 2006). This sequence contains two copies of *GADD45G*-like gene (LOC103130705 and LOC103130706). DNA-seq data of guppy *Aripo* female (ERX2156226) and male (ERX2156238) embryos (PRJEB22221 project database; Bergero *et al.* 2018) was downloaded and aligned to the *P. formosa* sequence using Gap5 software (Bonfield and Whitwham 2010). The annotated nucleotide sequence of this directed assembly of *GADD45G*-like, including the two orthologous genes present in *P. reticulata* locus is provided in Supplemental Figure S1.

### RNA seq analysis

The genomic guppy *GADD45G*-like sequence (LOC103474023) was BLAST searched using a word size of 48 bp against RNA-seq data (12 expression libraries) of female (SRX388849) and male (SRX388850) guppy embryos and organs (PRJNA230881 project database; Sharma *et al.* 2014). The resulting reads were aligned to reference transcripts of *P. formosa* genes (LOC103130705 and LOC103130706) using Gap5. The number of hits were compared between spliced isoforms, normalized and compared between males and females.

### Sex reversal treatment

Each female was introduced with a single male into a separate container. After the first spawn, each female was fed continuously with 30% protein pellet containing β-estradiol, at concentration of 200, 400 or 600 mg/kg, in order to induce female phenotype of fingerlings of subsequent spawns. All fries of second spawns were collected and reared separately until maturity while given dry feed containing β-estradiol continuously. Ultimately, individual fingerlings of 11 “sex reversed” families were identified for sex and fin clipped for DNA extraction.

### Histopathology

Histological analyses of 98 treated and control fish were examined of which 47 were sex reversed females. Fish were anesthetized, a cut in the abdomen was performed and the tail fin was cut off. The body was then immediately fixed in neutral buffered formalin and transferred to 70% ethanol after 48 h. Processing was performed after decalcification (44% formic acid and 12.5% sodium citrate for 12 h) using a microwave histo-processor (RHS-1, Milstone, Italy), after which the samples were embedded in paraffin blocks and sectioned at 4 μm. Sections were stained with hematoxylin and eosin (H&E). Samples were analyzed under a light microscope with 400× magnification for identification of the gonad and its condition thus determining the sex of the fish and the stage of sexual development.

### Data availability

Supplemental Table S1 contains the sequences of primers for 8 polymorphic markers on LG12. Supplemental Figure S1 contains the assembled genomic sequences of *GADD45G*-like gene locus of *P. reticulata*. Supplemental material available at FigShare: https://doi.org/10.25387/g3.9841160.

## Results

**Figure 1:**
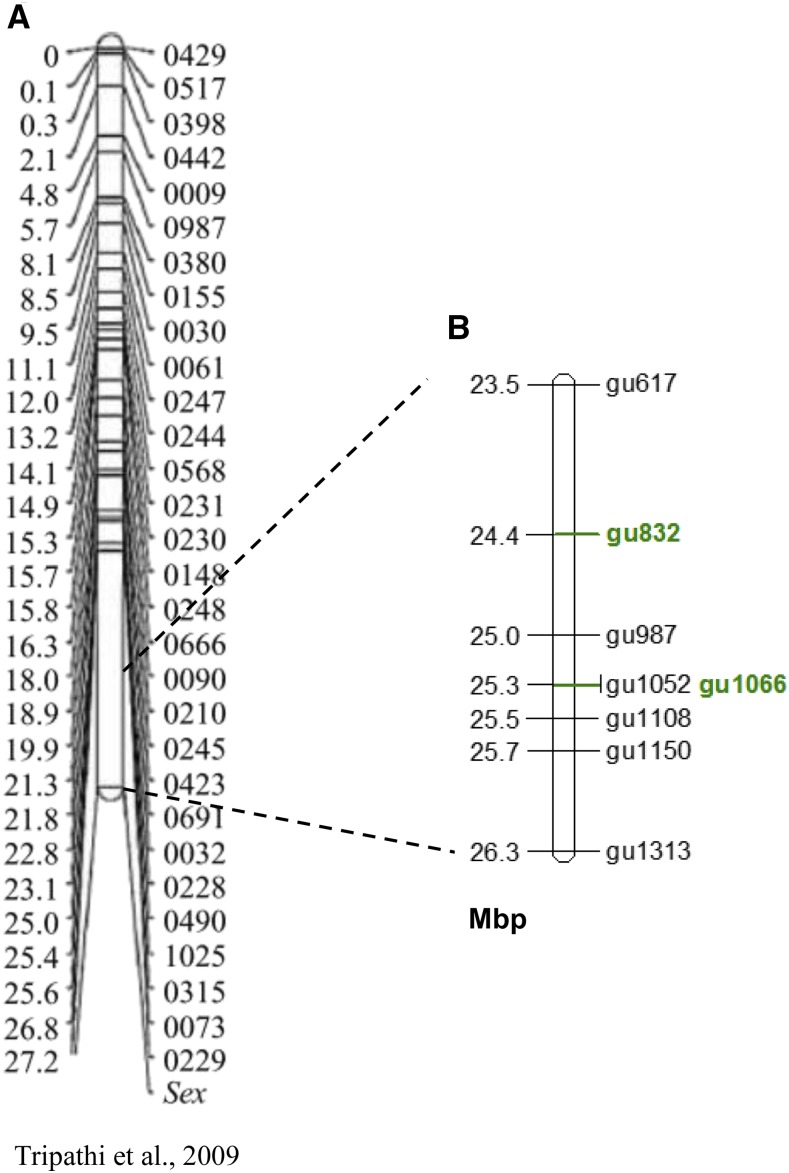
Mapping of sex determining region on LG12. A. Mapping of the sex region on LG12 in guppy as presented in Tripathi *et al.* (2009); B. Location of polymorphic microsatellites on LG12 terminal region, with the significant microsatellites associated with sex marked in green, Units are in Mbp.

**Figure 2:**
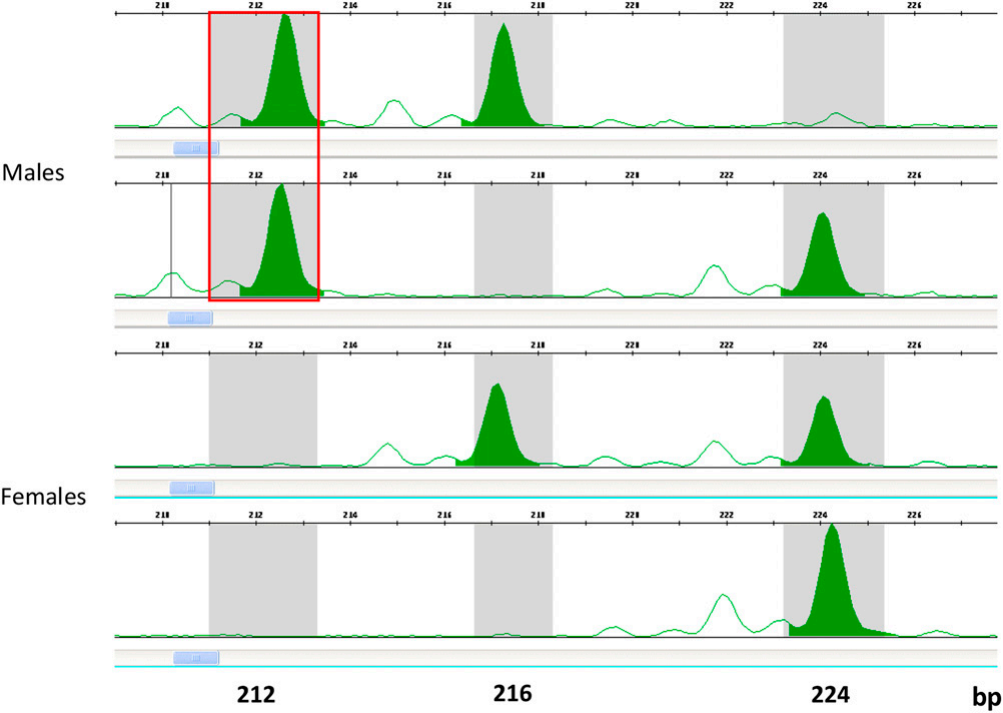
A typical genotyping results of two guppy males and two guppy females for microsatellite gu1066. Allele size is marked on the X-axis (in bp). The fluorescent intensity is marked on the Y-axis. Red square marks the male-specific allele representing “Y” (212 bp), whereas all other alleles represent “X” (216 or 224 bp).

Of 15 microsatellite markers designed in several locations along the terminal 5.4 Mbp genomic region on LG12, 7 were polymorphic in populations of Red Blonde and Flame with 2 to 6 alleles per marker. Five markers were polymorphic in progeny of 4 families with 2 to 4 alleles per marker. The locations of 8 polymorphic markers on LG12 in Mbp are displayed on Figure 1B, along with the previous map of the sex determining region on LG12 (Figure 1A). Association study for sex based on 46 individuals of Red Blonde and Flame strains showed complete concordance for only the gu1066 microsatellite (*P* < 5.7E-10, Table 1). Smaller batches of these populations and full-sib progeny showed lower significance in the association study. Nevertheless, a male-specific allele for gu1066 was evident in both strains (Table 1, Figure 2). The length of the male-specific chromosomal segment was 212 bp in all strains and families; as compared to either 206 or 212 bp for the Red Blonde batch in Manor Farm. In 21 out of 143 males (14.7%) the male-specific allele was not identified. Nevertheless, this allele was not found in any of the 132 tested females (Table 1). Thus, presence of the specific allele indicates male determination.

**Figure 3:**
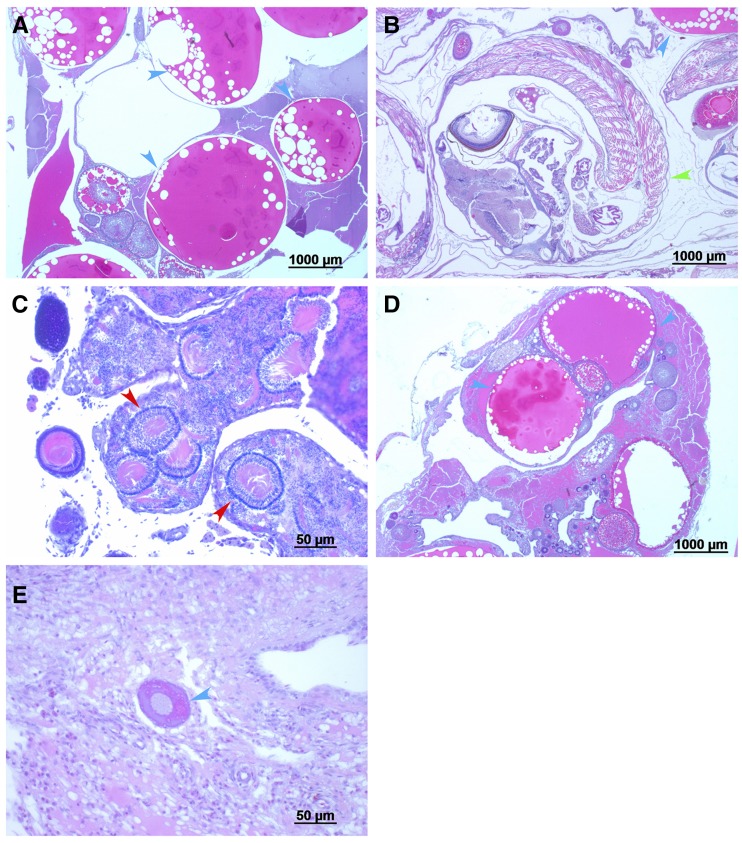
Histological examination of gonads of guppy at 10-14 months old. A. XX, female, normal ovarian tissue; B. XX, female with embryo; C. XY, male, normal testicular tissue; D. ΔXY, female, normal ovarian tissue; E. ΔXY, high proportion of undifferentiated tissue. XX and XY were determined based on genotype for gu1066 marker; Sex was defined by phenotype; Δ – β-estradiol treatment; Blue arrows – follicles in different developmental stage; Red arrows – seminiferous tubules; Green arrow - embryo. Size of ladder (in micrometer) is underlined in the lower right of each picture.

The distribution of males and females in sex reversed families (all-females) based on identification of the “Y” male-specific allele for microsatellite gu1066, is presented in Table 2. Non-significant deviation from a 1:1 ratio between females and males was evident in progeny of all families except one (Family #8). The twofold frequency of females as compared to males in this family with a nominal probability of significance of 0.03 is also non-significant when accounting for multiple comparisons (*P* = 0.33). Furthermore, based on the results in Table 1, the male-specific allele was identified in only 85% of males, but not in females. Thus, the observed overall distribution of 97 males and 129 females based on male-specific allele identification may be adjusted accordingly, resulting in similar proportions of males and females. Histological examination was performed on the ovaries of 47 sex reversed females and 25 genetic females. Typical histological analyses are presented in Figure 3 confirming the status of gonads. Of the sex reversed females, 19% exhibited definite matured ovaries, as compared to 64% in controls (Chisq; *P* < 8.9E-23).

**Table 2:** Distribution of genotypes in sex reversed families (all-females) based on detection of the “Y” male-specific allele of microsatellite gu1066

Family*^a^*	β-estradiol treatment*^b^*	XY	XX	N/A*^c^*
1	400	6	8	2
2	400	18	18	5
3	200	12	18	
4	400	12	13	
5	600	10	13	
6	200	6	8	
7	400	7	13	
8	600	5	10	
9	200	8	7	9
10	400	6	10	2
11	600	7	11	
Total		97	129	

a Deviation from equal distribution of males and females was not significant for all families with application of Bonferroni correction for multiple comparisons (*P* > 0.05).

b β-estradiol concentration in food (mg/kg).

c N/A – Not analyzed due to unsuccessful DNA extraction.

**Figure 4:**
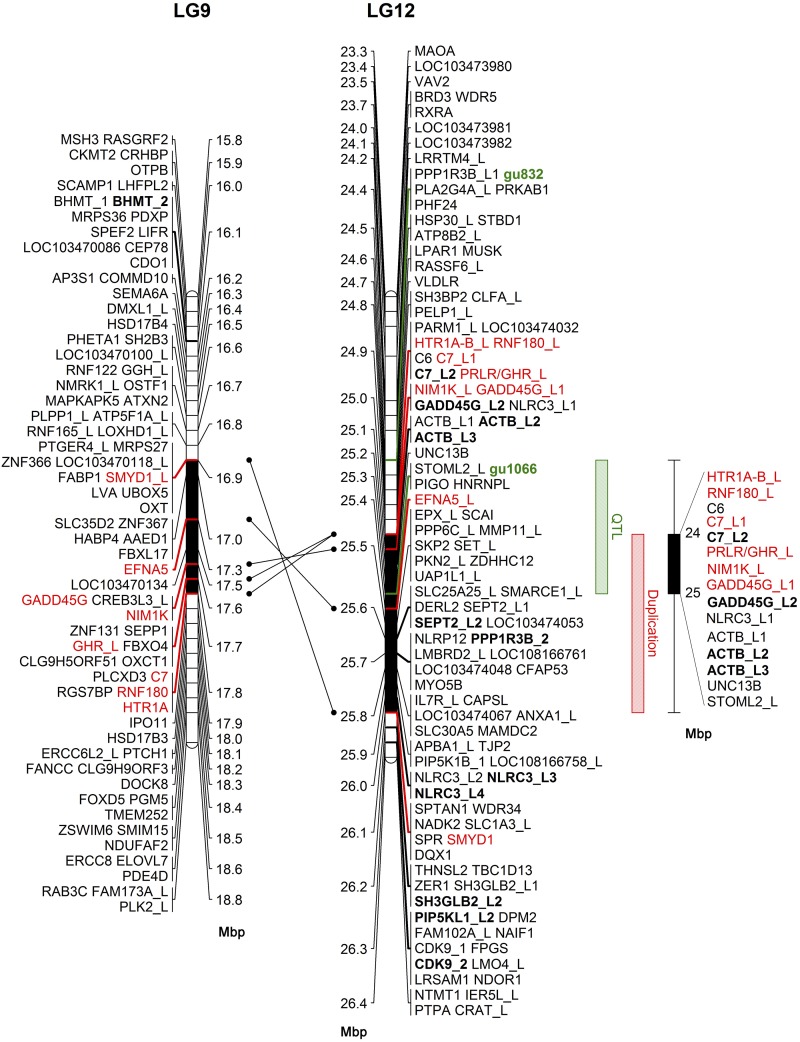
Comparative analysis of annotated genes between the critical SD region on LG12 and the respective duplication region on LG9 in guppy. Annotated genes are positioned in linkage groups 9 and 12. The region on LG12 between markers gu832 and gu1066 (in green) is represented by a green box on the right. Duplicated genes across LGs 9 and 12 are in red with connecting arrows between their respective positions. The common duplication region on both LGs is in black and represented by red box shape on the right. Annotated genes with multiple copies are marked with bold and underscore followed by the copy number. Gene name annotations containing “like” are marked with underscore followed by “L” and the copy number. Intersected region of the QTL and the duplication regions are represented by black box shape on the right with gene name annotations with copy numbers separated by “/”. Units are in Mbp.

Marker gu1066 at 25.3 Mbp was associated with sex in both strains (*P* < 5.7E-10, Table 1). Marker gu1066 is located within the Stomatin-like 2 gene *(STOML2*). In Significance values for association between gu1066 and adjacent segregating microsatellites are presented in Table 3. The upstream adjacent marker *e.g.*, gu1150, at 25.6 Mbp, was not associated with sex in both strains. However, the downstream adjacent marker gu832 at 24.4 Mbp, had the second most significant association with sex; based on the Flame strain (*P* < 3.9E-07). Thus, the genomic interval of 0.9 Mbp between gu832 and gu1066 markers, harboring 27 annotated genes, was selected for detailed analysis (Figure 4).

**Table 3:** Probability values of Fisher’s exact test for association between gu1066 and adjacent segregating microsatellites in the critical region on LG12 for sex determination

	Markers				
Line*^a^*	gu617 n(23.5)	gu832 (24.4)	gu1052 (25.25)	gu1108 (25.48)	gu1150 (25.6)
FL*^b^*	0.035	3.9E-07	0.046	NS*^c^*	NS
RB*^d^*	NS	NS	NS	NS	NS

a Genotypes for gu1066 had complete concordance with sex. Mapping position on LG12 for each microsatellite is shown in bracket (in Mbp).

b FL – Flame (n = 48).

c Not significant.

d RB – Red Blonde (n = 48).

In the search for enriched region with genes with multiple copies that may be associated with SD, we identified a region of duplication between LGs 9 and 12, of 1.26 Mbp, containing 59 genes on LG12 (Figure 4). In this region 17 genes had multiple copies (29%), of which 8 genes had copies across LGs 9 and 12 (in red), and the remaining 9 genes within LG12 (in bold). The common interval of the segment bordered by gu1066 and gu832, and the duplication region ranged from 24.88 to 25.31 (0.43 Mbp). This interval included 11 genes of which 6 (54%) *i.e.*, *HTR1A*, *RNF180*, *C7*, *GHR*, *NIM1K* and *GADD45G* like have multiple copies (black box shape, Figure 4).

**Figure 5:**
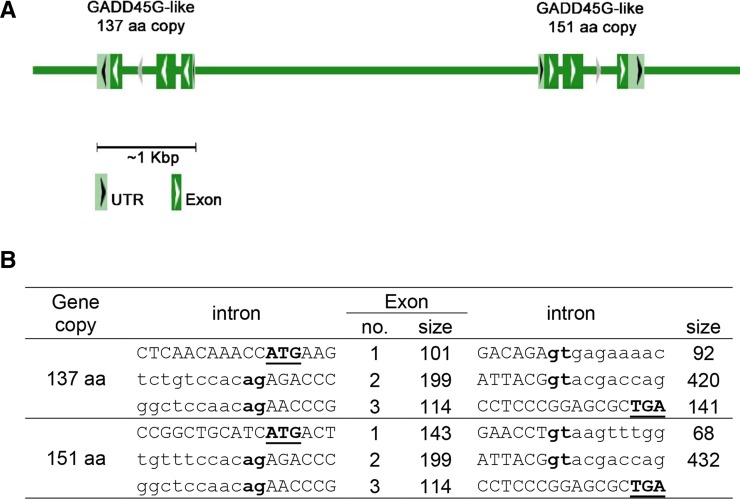
The structure of the duplicated *GADD45G*-Like gene in Guppy. The schematic (A) and genomic (B) structure of *GADD45G*-Like gene. Exon and intron sizes are given in base pairs. Intron and exon sequences are written in lowercase and uppercase letters, respectively. The first and last two bases of introns (gt and ag for donor and acceptor splice sites, respectively) are in bold type. The initiation and stop codons, and the putative poly adenylation signal (ATG, TGA) are in bold and underlined type.

The Growth arrest and DNA damage Inducible Gamma-like gene (*LOC103474023*, *GADD45G* like) is the most attractive candidate gene for SD in this region, as it has a specific role in male fertility and testis development (Johnen *et al.* 2013). This gene was prioritized for analysis. Assembly of 1428 reads located by BLAST-searching project PRJNA230881, indicated two *GADD45G*-like spliced isoforms of 151 and 137 amino acids that have not been characterized in *P. reticulata* but present in *P. formosa* (XP_007542235 and XP_007542234, respectively). Genomic sequences of both copies of the *GADD45G*-like gene in guppy, were assembled in a head-to-head orientation with a ∼4 Kbp intergenic region between them (Figure 5, Supplemental Figure S1). The two assembled copies contain 3 exons of which only the first one differed in sequence and size between them. The *GADD45G*-like genomic sequences of females and males were explored with no significant differences between them. Mean expression was 15 fold higher in the 151 *vs.* 137 AA isoforms based on 12 different expression libraries of guppy (Sharma *et al.* 2014). However, our analysis for *GADD45G*-like gene expression based on the latter data showed no definite sex-biased difference.

## Discussion

Eight of the 15 markers that were developed based on the published female guppy genome were found polymorphic with two or more alleles in Red Blonde and Flame strains and in full-sib families of Red Blonde. The association study for sex showed complete concordance for only the gu1066 microsatellite in both strains. Nevertheless, in additional small batches of samples the male-specific allele was not detected in all males, although not detected in any female. Failure to determine all males based on gu1066 genotype may be due to the occurrence of null alleles (Paetkau and Strobeck 1994), although design of alternative primers along the target sequence did not alter the genotypes (data not shown). Interestingly, additional markers that were mapped closely to gu1066, such as gu1108 and gu1150, based on the published female guppy genome, were not significantly associated to SD on LG12, possibly because of the difficulty to determine their position in duplicated regions. This ambiguity in precise locations of markers in relation to the sex region was also observed by Tripathi *et al.* (2009).

The second most significant associated marker in Flame, but not in the Red Blonde line, was gu832 located 0.9 Mbp from gu1066, thus validating the finding of a distal region on LG12 affecting sex determination (Wright *et al.* 2017), and some differences between Y chromosomes of different lines (Tripathi *et al.* 2009). A follow up analysis of full-sib progeny of the Red Blonde family D that segregated for both markers showed that two males out of 21 that lacked the 212 bp male-specific allele of gu1066, also lacked the 352 bp male-specific allele of gu832; thus indicating a possible occurrence of recombination outside the SD region harboring both markers and the sex determining locus. Nevertheless, these two males were progeny of a single family out of the four families tested (Family D), indicating a possible environmental effect of elevated temperature on sex reversal of these individuals (Karayucel *et al.* 2006). Moreover, none of the 132 tested females, including the 17 females in family D, displayed the male-specific allele as the putative reciprocal recombinant, in agreement with possible environmental effect or recombination suppression (Traut and Winking 2001; Tripathi *et al.* 2009; Charlesworth 2018). It may be possible that this type of recombinant is lethal, and thus was not found in adult females. Alternatively, the rare recombination may represent gene conversion that occurs in regions that do not undergo crossing over (Bergero *et al.* 2018; Bergero *et al.* 2019; Korunes and Noor 2017). Although sex is a categorical trait, a quantitative trait loci (QTL) analysis of the sex determining region may be more appropriate, accounting for aberrant crossing-over, segregation of genes in different regions and environmental effects (Eshel *et al.* 2010; Dor *et al.* 2016).

The chromosomal region of 0.9 Mbp between gu1066 and gu832 includes 27 annotated genes. We searched this region for enrichment of genes with multiple copies, because of the role of gene duplication in the turnover of sex chromosomes in teleosts (Eshel *et al.* 2014; Matsuda *et al.* 2002; Nanda *et al.* 2002; Hattori *et al.* 2012; Rondeau *et al.* 2016; Pan *et al.* 2019). We identified a region containing 59 genes on LG12 that was enriched with 17 genes (29%) with multiple copies, of which 8 had copies across both LGs 9 and 12. The length of the intersected interval between the two regions is 0.43 Mbp, containing 11 genes of which six have multiple copies. *GADD45G*-like is the most attractive candidate gene for SD in the intersected QTL/duplication region, as it has a specific role in male fertility and testis development (Johnen *et al.* 2013), and functions in male sex determination by promoting p38 signaling and SRY expression (Gierl *et al.* 2012). Furthermore, *GADD45G*−/− XY mice were born as completely sex-reversed XY-females due to their gonads failure to achieve the SRY expression threshold necessary for testis differentiation, resulting in ovary and Müllerian duct development (Johnen *et al.* 2013). We characterized the genomic structure of the gene in guppy, and found two isoforms that share identical sequence for exons 2 and 3, but not for exon 1 (Figure 5, Supplemental Figure S1). Mean expression was 15 fold higher in the 151 *vs.* 137 AA isoforms based on 12 different expression libraries of guppy (Sharma *et al.* 2014). However, we did not find any significant difference between sex groups in either genomic sequence or gene expression.

Forty putative Y genes, including two plausible candidate genes that may be involved in sex determination, were also suggested in guppies (Morris *et al.* 2018). To detect the progenitor of the guppy MSD gene on LG12, we analyzed the presumptive ancient region on LG9, and did not detect strong candidate genes for MSD, except for *GADD45G*. As the genome reference of a female guppy was used for assembly of genes, the MSD gene might presumably be missing in the SD region. However, high similarity was found between the genes on LGs 9 and 12. Moreover, the duplicated male-specific copy *of AMH* in tilapia is located in tandem along with the regular gene in female (Eshel *et al.* 2014; Li *et al.* 2015). Thus, it is implicated that the female guppy genome may contain the MSD gene and/or its regulatory elements.

Comparison of female and male synaptonemal complex showed that XY synapsis occurred rarely and preferentially in the terminal positions reflecting suppression of recombination over the entire Y chromosome, except a 1-2 Mbp of the small pseudo-autosomal region (Lisachov *et al.* 2015). A lack of crossing over in Y chromosomes over 25 Mbp region was supported by marker analysis for most guppy populations and families (Bergero *et al.* 2018; Bergero *et al.* 2019). However, Wright *et al.* (2017) predicted different patterns of recombination between X and Y over a 15-22 Mbp region of high recombination. In a preliminary study we analyzed sequence data of guppy family along three generations (GenBank project PRJEB7924), and found no crossing over in a 25 Mbp region of Y chromosome (data not shown). However, in the present study we detected significant level of recombination at an interval of 21-26.4 Mbp. Analysis of recombination in tilapia sex determining regions demonstrated that rate of recombination is highly affected by mating between closely related species (McConnell *et al.* 2000; Shirak *et al.* 2006; Cnaani *et al.* 2008). We hypothesize that several commercial, laboratory and natural stocks are hybrids of *P. reticulata* and related species. The mtDNA analysis of *P. wingei* natural population revealed its origin as hybrid with *P. reticulata* (Schories *et al.* 2009).

**Figure 6:**
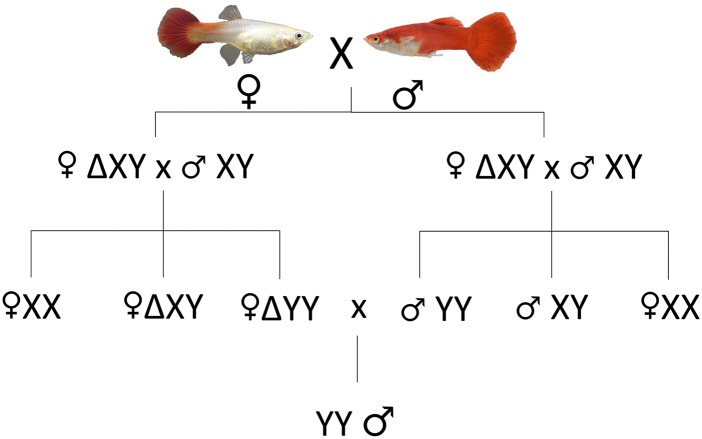
Mating scheme for production of all-males population based on detection of the “Y” male-specific allele of microsatellite gu1066. Δ – Sex reversed using β-estradiol, validated by SD test.

The incentive to mate sexually reversed females that are genetically males (XY) with normal males (XY) in order to derive YY males is dependent on the ability to genetically identify males using a DNA-based method. The entire scheme of mating is displayed in Figure 6. Subsequently, the YY males would be mated with normal females in order to produce XY fingerlings that will constitute the all-male commercial population (F3, Figure 6). Using the gu1066 marker we identified unequivocally 97 sex reversed females as genetically males, and used them for mating with normal males (F2, Figure 6). However, only 19% exhibited definite histologically matured ovaries, and only a single sex reversed female spawned four live fingerlings of which three were determined as XY and one as XX. Thus, further experiments are needed to assess the viability of YY genotypes of the Red Blonde and Flame strains, and the potential of this strategy to produce all-males population.
